# Dual stimuli-responsive polyurethane-based hydrogels as smart drug delivery carriers for the advanced treatment of chronic skin wounds

**DOI:** 10.1016/j.bioactmat.2021.01.003

**Published:** 2021-02-19

**Authors:** Rossella Laurano, Monica Boffito, Michela Abrami, Mario Grassi, Alice Zoso, Valeria Chiono, Gianluca Ciardelli

**Affiliations:** a Politecnico di Torino, Mechanical and Aerospace Engineering Department, Corso Duca degli Abruzzi 24, 10129, Torino, Italy; b Università degli Studi di Trieste, Department of Engineering and Architecture, Via Alfonso Valerio 6/1, 34127, Trieste, Italy

**Keywords:** Stimuli-responsive hydrogel, Drug delivery system, pH-triggered release, Advanced wound treatment, Polyurethane hydrogel, LF-NMR characterization

## Abstract

The design of multi-stimuli-responsive vehicles for the controlled and localized release of drugs is a challenging issue increasingly catching the attention of many research groups working on the advanced treatment of hard-to-close wounds. In this work, a thermo- and pH-responsive hydrogel (P-CHP407) was prepared from an *ad hoc* synthesized amphiphilic poly(ether urethane) (CHP407) exposing a significant amount of –COOH groups (8.8 ± 0.9 nmol/g_polymer_). The exposure of acid moieties in P-CHP407 hydrogel led to slightly lower initial gelation temperature (12.1 °C *vs.* 14.6 °C, respectively) and gelation rate than CHP407 hydrogel, as rheologically assessed. Nanoscale hydrogel characterization by Low Field NMR (LF-NMR) spectroscopy suggested that the presence of carboxylic groups in P-CHP407 caused the formation of bigger micelles with a thicker hydrated shell than CHP407 hydrogels, as further proved by Dynamic Light Scattering analyses. In addition, P-CHP407 hydrogel showed improved capability to change its internal pH compared to CHP407 one when incubated with an alkaline buffer (pH 8) (e.g., pH_change_5min_ = 3.76 and 1.32, respectively). Moreover, LF-NMR characterization suggested a stronger alkaline-pH-induced interaction of water molecules with micelles exposing –COOH groups. Lastly, the hydrogels were found biocompatible according to ISO 10993 and able to load and release Ibuprofen: delivery kinetics of Ibuprofen was enhanced by P-CHP407 hydrogels at alkaline pH, suggesting their potential use as smart delivery systems in the treatment of chronic infected wounds.

## Highlights

• Chronic infected wounds are characterized by the production of alkaline exudate.
• Multi-stimuli-responsive hydrogels are powerful tools to design smart drug carriers.
• Alkaline wound exudate can successfully guide drug release kinetics.
• Hydrogel thermosensitivity allows easy injectability in the wound site.
• LF-NMR describes nano-scale hydrogel structural changes in an alkaline environment.

## Graphical abstract

**Figure:**
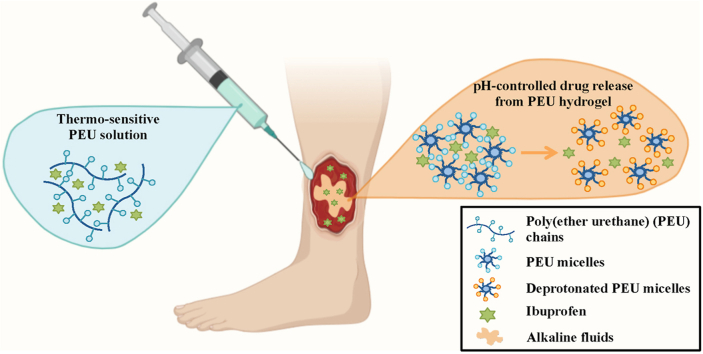

## 1 Introduction

The growing number of patients affected by hard-to-close wounds all over the world (4 million patients every year only in Europe) represents a severe burden for both the society and the healthcare systems, requiring long hospitalization and expensive treatments. Furthermore, patients suffering from impaired wound healing are forced to experience a low quality of life, due to difficulties in the management of pain associated to chronic wounds. Moreover, the oral administration of high doses of drugs (e.g., anti-inflammatory and analgesic drugs and/or antibiotics) required for their treatment may in turn cause side effects on not-target tissues (e.g., stomach, kidneys). Topical drug administration directly in the wound site could represent an effective strategy to maximize the success of the treatment and improve patients' comfort while minimizing the therapeutic dose and, eventually, side effects. In this regard, many works have been reported on the design of drug delivery systems for the *in situ* release of anti-inflammatory drugs [1,2], anti-microbial agents [3,4] and biomolecules [5,6] for a more effective treatment of hard-to-close wounds. However, such systems generally lack control over drug release kinetics, thus reducing the potentialities of the localized drug administration route. Among the wide plethora of available drug delivery systems (e.g., nanofibrous membranes, hydrogels, nanoparticles), stimuli-responsive hydrogels could be a powerful tool, overcoming the previously mentioned limitations proper of traditional wound cares. Stimuli-responsive hydrogels are three-dimensional highly hydrophilic networks able to modify their structure and properties in response to external stimuli, such as temperature [7], pH [8], UV/Vis light [9], electric field [10], magnetic field [11], chemical or biological agents [12]. Hence, these smart systems are attracting widespread interest in the design of drug delivery carriers showing responsiveness to specific stimuli, which allow a triggered payload release. In this context, exploiting the characteristic alkaline exudate of infected wounds [[13], [14], [15], [16], [17]] as triggering stimulus, this work was aimed at designing a dual stimuli-responsive hydrogel-based drug delivery system for the controlled release of encapsulated drugs in the advanced treatment of hard-to-close wounds. In this perspective, Ibuprofen was selected as anti-inflammatory and analgesic drug, being widely exploited for the treatment of hard-to-close wounds [[18], [19], [20]]. To overcome the huge efforts often required to properly design copolymer simultaneously responding to temperature and pH changes while maintaining water solubility and cytocompatibility [[21], [22], [23], [24], [25]], in this work the LEGO-like poly(urethane) chemistry has been successfully explored to engineer hydrogel constituent materials. Recently, Laurano and Boffito et al. [26] proposed an innovative and easy method to prepare a water-soluble thermo- and pH-responsive hydrogel from a synthesized amphiphilic poly(ether urethane) (PEU), functionalized with carboxylic acid groups-containing chains. Specifically, the PEU was synthesized starting from the commercially available triblock copolymer Poloxamer® 407, an aliphatic diisocyanate (i.e., 1,6 hexamethylene diisocyanate) and a cyclic chain extender (i.e., 1,4-cycloexhanedimethanol). Then, plasma treatment was applied on PEU powders and parameters were optimized to maximize the number of exposed carboxylic acid groups while avoiding polymer degradation. An in-depth investigation of polymer thermo-responsiveness after powder plasma treatment was carried out through the estimation of the temperature at which polymeric chains started to arrange into organized micelles. On the other hand, expected pH-responsiveness, attributed to grafted/polymerized acidic groups, has not been investigated up to now. Therefore, starting from the previously developed polymer, in this work a thorough investigation of the influence of carboxylic acid group exposure on hydrogel gelation kinetics and timing was carried out by tube inverting test and rheological characterization. On the other hand, hydrogel alkaline pH-responsiveness was studied by measuring its capability to transfer pH changes from the surrounding medium to its core and through swelling/stability tests in contact with buffers at different pH values. Furthermore, system performances in terms of temperature-driven hydrogel formation mechanism and pH-dependent structural changes were also investigated at the nano-scale through Low Field Nuclear Magnetic Resonance (LF-NMR) spectroscopy. Indeed, by exploiting the property of water hydrogen atoms to be permanent dipoles, this technique allows the measurement of the spin-spin relaxation times T_2i_, i.e., the time that water hydrogens require to recover their initial orientation after the perturbation induced by an applied magnetic field. The identification of multiple T_2i_ reflects the presence of water in different forms (e.g., free or bound water) and thus, gives information on the interactions occurring between water molecules and polymeric chains. Due to the high versatility of this technique, LF-NMR has found application also in the characterization of hydrogel-based drug delivery systems to predict payload release kinetics through the measurement of the mesh size and crosslinking degree. For instance, De’ Nobili et al. [27] combined rheology and LF-NMR characterization to determine the average network mesh size of pectin and pectin-alginate films loaded with L-(+)-ascorbic acid. Kirchhof et al. [28] investigated the relationship between mesh size and release rate of both a model drug (i.e., fluorescein isothiocyanate dextran) and an antibody from hydrogels cross-linked via Diels-Alder reactions. Finally, Abrami et al. [29] studied the structure of cross-linked hydrogels based on alginate and Poloxamer® 407, highlighting the presence of small and big meshes inside them, accounting for a double-release kinetics. However, in all these previous studies, hydrogels formed physical or chemical cross-linked networks. In this work, to the best of our knowledge, LF-NMR was applied for the first time to study the stimuli-responsiveness of micellar hydrogels. Finally, the potentiality of the here-developed hydrogel to be successfully applied as delivery system in the treatment of chronic wounds was assessed through drug release tests conducted in pathological-mimicking conditions (i.e., by incubating the gels in an alkaline buffer) and analyzed by applying the Korsmeyer-Peppas model.

## 2 Materials and methods

### 2.1 Polymer synthesis and powder plasma treatment

The P-CHP407 polymer used in this work resulted from an optimization of the powder plasma treatment carried out in a recently published work by Laurano and Boffito et al. [26] to maximize the amount of exposed –COOH groups on a water-soluble polymer while preventing its degradation. Briefly, the Poloxamer®-based PEU was first synthesized following a two-step procedure under inert atmosphere. In the first step, the macrodiol Poloxamer® 407 (20% w/V - P407, Sigma Aldrich, Italy) was reacted for 150 min at 80 °C in anhydrous 1,2-dichloroethane (DCE, Carlo Erba Reagents, Italy) with 1,6-hexamethylene diisocyanate (HDI, Sigma Aldrich, Italy) in a 1:2 molar ratio and in the presence of a catalytic amount of dibutyltin dilaurate (0.1% w/w - DBTDL, Sigma Aldrich, Italy). Then, the isocyanate-terminated prepolymers were chain extended for 90 min at 60 °C upon the addition of 1,4-cyclohexanedimethanol (CDM, Sigma Aldrich, Italy) at 1:1 molar ratio. Finally, the reaction was terminated by adding anhydrous methanol (Sigma Aldrich, Italy). The high molecular weight PEU, referred to with the acronym CHP407, was collected by precipitation in petroleum ether (4:1 V/V with respect to DCE volume). Subsequently, CHP407 (20% w/V) was dissolved in DCE and purified by precipitation in a mixture of diethyl ether/methanol (98/2 V/V) at 5:1 V/V with respect to DCE volume. Lastly, CHP407 was collected by centrifugation (Hettich, MIKRO 220 R) at 0 °C, 6000 rpm, for 20 min and dried overnight under the fume-hood.

Subsequently, CHP407 was ground and sieved to produce powders (5 g) with controlled average diameter (<500 μm). Plasma treatment was then carried out in a two-step functionalization procedure using a plasma reactor (Diener Electronic, Germany) equipped with a rotary system. Specifically, powder surface activation was performed by applying a direct power at 50 W for 5 min; then, acrylic acid (AA) vapor grafting/polymerization on activated surfaces was conducted by applying an alternated power at 200 W (9 Hz, Pulse ON 10 ms, Pulse OFF 1000 ms, Duty Cycle 0.01, mean power applied 22 W) for 10 min upon filling the reactor chamber with AA at 10 μL/min for 15 min. The Ar gas flow rate was kept constant at 10 sccm during the entire process. Subsequently, plasma-treated PEU powders (2.5% w/V), referred to with the acronym P-CHP407, were dissolved in chloroform and precipitated in petroleum ether (5:1 V/V with respect to chloroform) to remove unreacted AA. Finally, the collected P-CHP407 was dried under the fume-hood and stored under nitrogen until use.

### 2.2 Poly(ether urethane) chemical characterization

To assess the success of the synthesis and to verify the absence of by-product formation during plasma treatment, both CHP407 and P-CHP407 were first characterized through Infrared spectroscopy and Size Exclusion Chromatography (SEC). Subsequently, the colorimetric Toluidine Blue O assay was performed on plasma-treated polymer and on CHP407 as control condition to quantify the exposed carboxylic acid groups. All characterizations were carried out according to the protocols published by Laurano and Boffito et al. [26] (see Supporting Information file for a detailed description of protocols).

### 2.3 Hydrogel preparation

CHP407- and P-CHP407-based hydrogels were prepared by dissolving the polymers at predefined concentrations (ranging between 5% w/V and 25% w/V) in physiological saline solution (0.9 g of NaCl in 100 mL of double demineralized water - ddH_2_O). To avoid micellization and/or gelation phenomena, which would prevent complete polymer dissolution, samples were kept at 4 °C overnight to achieve a complete polymer solubilization.

### 2.4 Tube inverting test

To qualitatively determine the Critical Gelation Concentration (CGC) of CHP407- and P-CHP407-based solutions and to evaluate their sol-to-gel transition temperature, Tube Inverting Test was performed on both CHP407 and P-CHP407 systems. Samples were prepared in bijou sample containers with an inner diameter of 17 mm (Carlo Erba Reagents, Italy) according to the previously described protocol and subjected to a controlled heating from 5 °C to 70 °C. Each step consisted of a 1 ± 0.1 °C temperature increase within 30 s followed by temperature maintenance for 5 min and tube inversion to visually inspect the sol-to-gel transition. Sol and gel conditions were defined as reported in Pontremoli and Boffito et al. [30]. Briefly, “flow liquid sol” and “no flow solid gel” within 30 s characterized the sol and gel states, respectively. Result variability ascribed to the operator's perception of the sol and gel states was assessed by asking to three different operators to perform the same test independently. Results were reported as mean ± standard deviation.

### 2.5 Gelation time at physiological temperature

The time required by P-CHP407-based solutions to undergo sol-to-gel transition at physiological temperature was estimated through gelation time test at 37 °C and compared to CHP407-based systems with the same compositions. Specifically, samples were prepared as for Tube Inverting Test and put in an incubator (Memmert IF75) equilibrated at 37 °C. Then, at predefined time points (1–10 min, 1 min/step) samples were taken from the incubator and inverted for 30 s for the visual inspection of their sol/gel state. Conditions of sol and gel were defined as in the Tube Inverting Test. At each time point, the samples were equilibrated at 4 °C for 8 min before incubation at 37 °C to ensure that all systems were in the sol state at the beginning of the test. Result variability ascribed to the operator's perception of the sol and gel states was assessed by asking to three different operators to perform the same test independently. Results were reported as mean ± standard deviation.

### 2.6 Rheological characterization

PEU-based hydrogels were rheologically characterized using a stress-controlled rheometer (MCR302, Anton Paar GmbH, Graz, Austria) equipped with a 50 mm parallel plate geometry and a Peltier system for temperature control. Temperature ramp tests were carried out (0 °C–40 °C, 2 °C/min, deformation rate 0.1 s^−1^) to study the sol-to-gel transition. Gel response to applied deformation was evaluated at 37 °C through strain sweep tests (10 Hz, strain range 0.01–500%), while gel viscoelastic properties were investigated by small amplitude oscillatory shear tests (frequency sweep tests performed within the hydrogel linear viscoelastic region, frequency range 0.1–100 rad/s, temperature 25 °C, 30 °C and 37 °C). Samples were prepared as previously described by dissolving the polymer (15% w/V) in physiological saline solution. Before each analysis, the sample was poured on the lower plate of the instrument in the sol state at 0 °C, heated at the selected temperature, kept in quiescent condition for 10 min to reach thermal stability and then tested.

### 2.7 pH variation in contact with buffers

To study hydrogel ability to transfer alkaline pH from the surrounding environment to their core, gel pH-sensitivity was evaluated by placing hydrogels in contact with buffers at pH 5 and 8 for predefined time intervals. In detail, CHP407- and P-CHP407-based systems (15% w/V) were first dissolved in ddH_2_O to avoid potential contributions in pH changes induced by the solvent and equilibrated at 37 °C for 15 min to allow a complete sol-to-gel transition. Subsequently, 1 mL of 0.5 M phosphate buffer solution at pH 5 or 8 (equilibrated at 37 °C to avoid hydrogel destabilization) was added upon each gel. At predefined time steps (5 min, 10 min, 15 min, 30 min, 1 h, 3 h, 5 h and 24 h), three samples were taken from the incubator and the residual buffer was removed. Then, samples were kept at 5 °C until complete gel-to-sol transition and the pH was measured using a pHmeter (pH 110, Eutech Instrument). Results were reported as mean ± standard deviation.

### 2.8 Hydrogel behavior in contact with buffers

Stability tests were carried out on CHP407 and P-CHP407 hydrogels (15% w/V) prepared according to the previously described protocol. Samples were first weighed to record their initial weight (W_i_) and then incubated at 37 °C for 15 min to allow a complete sol-to-gel transition. Subsequently, 1 mL of 0.5 M phosphate buffer solution at pH 5 or 8 (equilibrated at 37 °C to avoid gel destabilization) was added to each sample and incubated for 6 h, 1 day, 2 days, 3 days, 7 days and 14 days (complete medium refresh was performed every other day). At each time step, three samples were taken, the residual buffer was removed, and the hydrogels were weighed (W_f_) to assess their fluid absorption ability (Apparent Swelling %). Then, the samples were freeze-dried and weighed again (W_freeze-dried_f_) to quantify their weight loss (Weight loss %). Control samples (not incubated in buffer solutions) were also freeze-dried and weighed (W_freeze-dried_i_). Buffer absorption and system dissolution in aqueous environment were estimated according to equations published by Boffito et al. [31]. Furthermore, the potential occurrence of polymer degradation phenomena was assessed through SEC analyses performed at each time step on lyophilized samples according to the method published by Laurano and Boffito et al. [26].

### 2.9 Low Field Nuclear Magnetic Resonance (LF-NMR) spectroscopy

LF-NMR characterization was performed on both CHP407 and P-CHP407 hydrogels using a Bruker Minispec mq20 (0.47T, Germany). To investigate hydrogel gelation mechanism and kinetics and their pH transmission ability from the surrounding environment to their core, the spin-spin relaxation times T_2_ of PEU-based systems were measured according to the Carr-Purcell-Meiboom-Gill sequence with a 90°–180° pulse separation of 0.25 ms (number of scans = 4, delay = 5 s).

Specifically, to evaluate hydrogel thermo-sensitivity, the samples (500 μL, 15% w/V polymer concentration) were prepared in NMR glass tubes (diameter 8 mm) and stored at 4 °C until use. Then, the samples were incubated at 37 °C and the T_2_ value was recorded at 0 min, 4 min, 6 min, 8 min and 10 min. CHP407 samples were prepared and analyzed according to the same protocol as control condition.

For what concerns hydrogel pH-responsiveness, samples were prepared in NMR glass tube, maintained at 37 °C for 10 min to ensure a complete sol-to-gel transition and then put in contact with 500 μL of phosphate buffer solution at pH 5 or 8 (equilibrated at 37 °C) for 10 min, 20 min, 18 h and 24 h. Hydrogels prepared according to the same protocol and not incubated with buffer solutions were also analyzed and their T_2_ values were used as time zero data. At each time point, the residual buffer solution was removed and then the T_2_ value was acquired as previously described. Subsequently, for both the experiments, T_2_ discrete distribution was obtained following the protocol published by Marizza et al. [32].

### 2.10 pH-triggered drug release

To investigate hydrogel capability to release encapsulated drugs via a pH-controlled mechanism, drug release tests were conducted on both CHP407- and P-CHP407-based systems in contact with phosphate buffers at different pH values (i.e., 5 and 8 to simulate inflammatory and infected environment, respectively). Ibuprofen (IBU - Sigma Aldrich, Italy), selected as anti-inflammatory and analgesic model drug, was first dissolved in ethanol at 40 mg/mL [7]. Then, an aliquot of this solution was added to each higher-concentrated sample to reach a final IBU concentration of 1 mg/mL and the desired PEU concentration (i.e., 15% w/V). Subsequently, CHP407- and P-CHP407 hydrogels were incubated at 37 °C for 15 min to ensure a complete sol-to-gel transition and 1 mL of phosphate buffer at pH 5 or 8 was added upon each sample as release medium (IBU solubility at 37 °C was reported to be 0.28 mg/mL and 3.51 mg/mL at pH 5 and 8, respectively [33]). Extracts were collected and completely refreshed at different time points (i.e., 15 min, 30 min, 45 min, 60 min, 90 min, 2 h, 3 h, 4 h, 5 h, 7 h and 24 h). Released IBU quantification was carried out using a High Performance Liquid Chromatography (HPLC, Thermo Scientific, Dionex Ultimate 3000) instrument equipped with a C18 column (5 μm, 120 Å) [34]. Acetonitrile (ACN – Carlo Erba Reagents, Italy, HPLC grade) and phosphoric acid solution (0.03% w/V) were mixed at 60/40 V/V and used as mobile phase by setting 1.7 mL/min as flow rate. Before the analyses, 400 μL of each extract were diluted in 600 μL of ACN (to ensure complete IBU dissolution in the mobile phase) obtaining a final buffer/ACN volume ratio of 40/60. Then, the resultant sample was filtered through a 0.45 μm syringe filter (Macherey-Nagel, poly(tetrafluoroethylene) membrane, Carlo Erba Reagents, Italy) and analyses were carried out at room temperature for 5 min with an injection volume of 20 μL. Subsequently, the drug was quantified by referring to a calibration curve based on IBU standards with concentration in the range 0.05–1 mg/mL and by considering the area subtended by the peak at 214 nm. Lastly, to better understand whether different release mechanisms occurred in the presence of exposed carboxylic acid groups in P-CHP407 gels, the Korsmeyer-Peppas model was applied to release data and the drug transport mechanism was defined based on the estimated release exponent *n* value [35].

### 2.11 *In vitro* hydrogel cytocompatibility evaluation

To assess hydrogel cytocompatibility, cell viability tests were conducted on both CHP407 and P-CHP407 extracts prepared according to the ISO 10993 regulation. Specifically, 400 μL of hydrogel solution (15% w/V in physiological saline solution) were poured in a 24-well transwell and incubated at 37 °C for 10 min to allow a complete sol-to-gel transition. Subsequently, 400 μL of complete medium, previously equilibrated at 37 °C, were added to each well and samples were maintained at 37 °C for 24 h. Extracts were then collected and filtered through a 0.22 μm syringe filter (Polyethersulfone membrane, Carlo Erba Reagents, Italy) under a sterile hood. Meanwhile, NIH-3T3 murine fibroblasts were seeded in a tissue culture 96-well plate at a cell density of 62000 cells/cm^2^ in Dulbecco's Modified Eagle's Medium (DMEM – Thermo Fisher Scientific, Italy) supplemented with 10% Foetal Bovine Serum (FBS - Thermo Fisher Scientific, Italy) and 1% Penicillin/Streptomycin (Carlo Erba Reagents, Italy), and maintained in a humidified incubator at 37 °C, 5% CO_2_. 24 h after seeding, extracts were added to NIH-3T3 culture. After 24 h incubation, the medium was removed to proceed with viability assay. Briefly, Resazurin (Sigma Aldrich, Italy) was dissolved at 1 mg/mL in ddH_2_O, diluted 1:10 in complete medium and added to the cell culture plate after extracts removal. Cells were incubated at 37 °C, 5% CO_2_ for 1 h and fluorescence was measured with a plate reader (PerkinElmer, Victor X3) at ex/em = 530/590 nm.

### 2.12 Statistical analysis

Statistical analysis was performed using GraphPad Prism 8.0 for MacOsX (GraphPad Software, La Jolla, CA, USA; www.graphpad.com). Two-way ANOVA analysis followed by Bonferroni's multiple comparison test was used to compare results. The statistical significance of each comparison was assessed as reported in Boffito et al. [31]. Results are reported as mean ± standard deviation.

## 3 Results and discussion

### 3.1 Poly(ether urethane) characterization

A detailed physico-chemical characterization of the materials used in this work was reported in the Supporting Information file. Briefly, Infrared spectroscopic analysis conducted on P407 macrodiol (as control) and on the synthesized poly(ether urethane) (PEU) (CHP407) proved the successful synthesis of a P407-based PEU, as assessed through the concurrent appearance of urethane and P407 characteristic bands [26] (Fig. S1). The same analysis performed on P-CHP407 demonstrated the absence of vibrational bands attributed to plasma treatment by-products (Fig. S2). Furthermore, chromatographic analyses performed on CHP407 and P-CHP407 polymers confirmed the successful synthesis of a high molecular weight PEU (Number Average Molecular Weight ${\bar{M}}_{n}$ 30 kDa) and the absence of degradation phenomena ascribed to plasma treatment (Fig. S3).

Finally, the exposed carboxylic acid groups along P-CHP407 chains were quantified through Toluidine Blue O colorimetric assay, resulting to be 8.8 ± 0.9 nmol/g of P-CHP407 (Fig. S4).

### 3.2 Hydrogel characterization at the macro-scale through conventional techniques

To assess hydrogel thermo-sensitivity, ensured by the presence of Poloxamer® 407 as PEU building block, and pH-responsiveness to alkaline environment provided by carboxylic acid group exposure, CHP407- and P-CHP407 gels were first characterized through conventional techniques.

#### 3.2.1 Tube inverting and gelation time tests

**Fig. 1:**
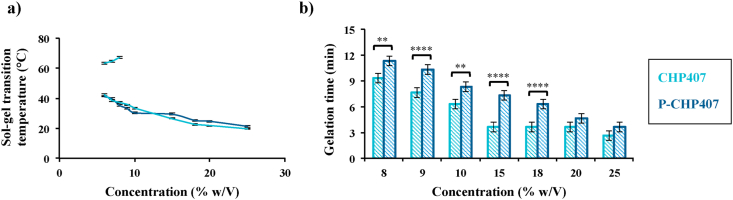
a) Tube Inverting Test conducted on CHP407 and P-CHP407 hydrogels prepared by dissolving the polymer in physiological saline solution at different concentrations (5%–25% w/V) within 5 °C–70 °C range (1 °C/step, systematic error ± 0.5 °C). The statistical analysis conducted to compare results from CHP407- and P-CHP407-based systems gave *p* values in the range 0.05–0.01, 0.01-0.001, 0.001-0.0001 for 25% w/V, 8% w/V and 9% w/V systems, respectively, while *p* values < 0.0001 were obtained for formulations with concentrations ranging between 10% and 20% w/V. b) Gelation time test at physiological temperature (1 min/step, systematic error ± 30 s) performed on CHP407 and P-CHP407 hydrogels prepared by dissolving the polymer in physiological saline solution at different concentrations (5%–25% w/V). Analyses were performed by three operators independently and results reported as mean ± standard deviation.

Hydrogel capability to undergo a sol-to-gel transition in response to temperature increase was first qualitatively studied through the Tube Inverting test. Moreover, this test also allowed the definition of the Critical Gelation Concentration (CGC), i.e., the lowest polymer concentration able to form a gel by temperature increase (Fig. 1a). On the other hand, the Gelation Time test conducted at 37 °C gave information about hydrogel gelation timing at physiological temperature (Fig. 1b). More in detail, these qualitative tests were conducted by dissolving both CHP407 and P-CHP407 polymers in unbuffered saline solution, meaning that two parameters were simultaneously considered, i.e., the polymeric concentration and solution pH (dependent on the amount of deprotonated –COOH groups). However, such testing conditions where on purpose selected to investigate the influence of carboxylic acid groups on the gelation process of un-treated and plasma-treated formulations prepared at the same polymeric concentration.

As illustrated in Fig. 1a, the gelation temperature of both CHP407 and P-CHP407 formulations was strongly dependent on their polymeric concentration, according to data reported in a previous work on similar PEU-based systems (i.e., PEUs differing for their chain-extender) [31]. Specifically, upon a decrease in PEU concentration a significant increase in the system gelation temperature was observed, irrespective of the tested polymer. Furthermore, the introduction of carboxylic acid groups through plasma treatment did not affect the sol-to-gel transition capability, as already suggested by previously reported results on the critical micellar temperature of these polymers [26]. Slightly different CGCs were measured for the systems: 6% w/V and 7% w/V (error ± 0.5% w/V) for CHP407 and P-CHP407 formulations, respectively. This difference, although low, could be attributed to the exposed hydrophilic functional groups on P-CHP407 chains, which contributed to increase the hydrophilic counterpart of the system, thus slightly lowering hydrogel capability to undergo micellization. Moreover, conversely to CHP407, P-CHP407 gels were not able to undergo a gel-to-sol transition upon further temperature increase (i.e., at temperatures higher than 60 °C P-CHP407 and CHP407 systems were present in the gel and sol phase, respectively), suggesting a higher thermal stability of P-CHP407 gel network.

Concerning hydrogel gelation kinetics at 37 °C (Fig. 1b), both systems showed a decrease in gelation time as a function of polymer concentration. However, at each polymer concentration, P-CHP407 solutions needed more time to complete gelation (e.g., for 15% w/V polymer concentration, gelation time at 37 °C was 3.7 ± 0.6 min for CHP407 and 7.3 ± 0.6 min for P-CHP407 hydrogels). These results further supported the hypothesis that the introduction of –COOH groups along the polymer backbone increased the hydrophilicity of the chains, thus slightly affecting the gelation parameters of P-CHP407 solutions respect to CHP407 ones.

Based on these results, 15% w/V was selected as a promising solution concentration for both systems, allowing gelation at approx. physiological temperature within few minutes (Fig. S5). Moreover, based on previous results [30], such composition was also easily injectable through G22 needles at 4 °C (i.e., in the sol phase) and through G18 needles up to room temperature (i.e., semi-gel state), according to the application hydrogels were designed for. Hence, further characterizations were performed on CHP407 and P-CHP407 hydrogels with this composition.

#### 3.2.2 Rheological characterization

P-CHP407 hydrogel gelation mechanism and kinetics were thoroughly investigated through temperature ramp test within the 0 °C–40 °C range, strain sweep test at physiological temperature and frequency sweep tests at 25 °C, 30 °C and 37 °C and results compared to CHP407 sol-gel systems.

**Fig. 2:**
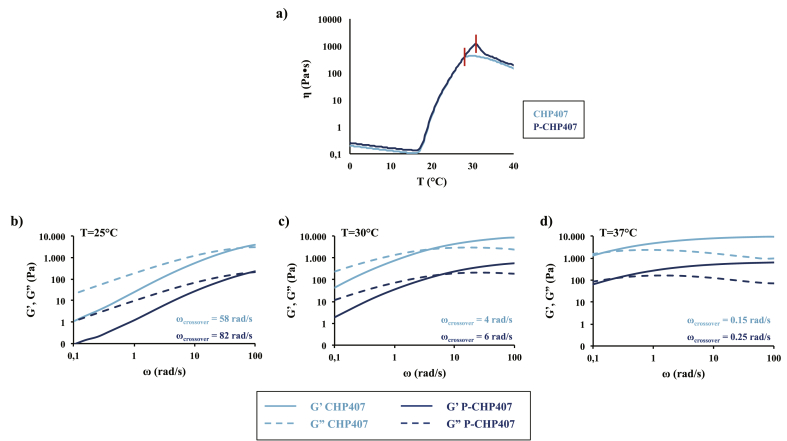
a) Viscosity (η) profiles *versus* temperature (T) during the sol-to-gel transition as analyzed through temperature ramp test performed on CHP407 and P-CHP407 solutions (15% w/V concentration) within 0 °C–40 °C temperature range. The red bar marks the appearance of micelle disentanglements leading to gel fracture. b), c) and d) Storage (G′) and loss (G″) moduli registered as a function of angular frequency (ω) within 0.1 rad/s and 100 rad/s for CHP407 and P-CHP407 (both G′ and G″ were divided by a factor 10) formulations (15% w/V concentration) at 25 °C, 30 °C and 37 °C. In each figure, the angular frequency value at the crossover point between G′ and G’’ (ω_crossover_) is reported for both CHP407 (dark blue) and P-CHP407 (light blue) formulations.

As illustrated in Fig. 2a, an initial temperature-driven viscosity decrease was registered for both the analyzed samples, reaching a minimum value that was measured to be 0.3 and 0.26 Pa∙s for CHP407 and P-CHP407 solutions, respectively. The temperature at the minimum of viscosity was considered the starting temperature (*T*_onset_) of the gelation process and was measured to be slightly lower for P-CHP407 system compared to CHP407-based one (12.1 °C *vs.* 14.6 °C, respectively). This result indicated that the critical micellar volume of 0.53 required for the onset of the sol-to-gel transition [31] was reached at slightly lower temperatures for P-CHP407 hydrogel compared to CHP407 one with the same concentration. Indeed, the exposure of carboxylic groups in P-CHP407 induced the nucleation of bigger micelles (see Supporting Information for Dynamic Light Scattering measurements) as a consequence of the interactions between –COOH groups and water molecules, with the formation of a hydrated shell. Subsequently, a sharp viscosity increase was registered, without differences between the samples, due to micelle nucleation, growth and progressive packing, with conversion of the sol into a biphasic system. Finally, viscosity reached a maximum value, followed by a decrease with further increasing temperature, ascribed to the onset of entropically favored and progressive micelle disentanglements finally leading to a complete gel fracture. However, the slightly higher temperature at which the viscosity of P-CHP407 formulation began to decrease (i.e., 30 °C for P-CHP407 *vs.* 27 °C for CHP407) could be attributed to a different degree of gel development. Specifically, the exposure of hydrophilic -COOH groups, which more strongly interacted with the surrounding water molecules, probably slightly hindered micelle aggregation, thus requiring higher temperature to complete hydrogel development. These results were in agreement with gelation time test data, which indicated a slower gelation kinetics for P-CHP407 (Fig. 1b).

To study the influence of exposed –COOH groups on gel formation upon temperature increase, frequency sweep tests were performed on both CHP407 and P-CHP407 sol-gel systems at 25 °C, 30 °C and 37 °C (Fig. 2b–d). Conventionally, the sol phase is characterized by Storage Modulus (G′) values lower than Loss Modulus (G″) ones. On the contrary, when G′ values are at least one order of magnitude higher than G″ ones and both moduli are frequency-independent, the system is considered to be in the gel phase. Based on these definitions, both systems turned out to be in the sol state at 25 °C, in a transition phase at 30 °C and in an incipient gel condition at 37 °C. The frequency at which G′ becomes greater than G” (ω_crossover_) is a peculiar parameter which marks the transition from viscous to elastic behavior. At each analyzed temperature, the G’/G″ crossover frequency of P-CHP407 formulations was slightly higher compared to CHP407, suggesting longer gelation time compared to CHP407 gels.

Hence, although the *T*_onset_ of P-CHP407 samples was slightly lower than for CHP407, meaning that the gelation process began at lower temperatures in the presence of –COOH groups (Fig. 2a), P-CHP407 gelation process was slower at each tested temperature. These results could be interpreted considering that the exposure of –COOH groups induced the formation of P-CHP407 micelles with larger size, which consequently required more time for their arrangement in an organized network.

Lastly, strain sweep tests (Fig. S7) performed on CHP407 and P-CHP407 gels reported similar trends of storage (7.9 kPa for CHP407 and 5 kPa for P-CHP407) and loss (1.6 kPa for CHP407 and 1.2 kPa for P-CHP407) moduli within the linear viscoelastic range. However, P-CHP407 gel showed a significantly higher critical deformation compared to CHP407-based sample (strain at breaking 29.7% *vs.* 18.6%). As at 37 °C both systems were in an incipient gel state (ω_crossover_ was 0.15 rad/s and 0.25 rad/s for CHP407 and P-CHP407 gels, respectively (Fig. 2d)), this result could not be exclusively correlated to a lower degree of P-CHP407 gel development with respect to the control, but rather to the stronger and hydrogen bond-mediated micelle-micelle interactions occurring in P-CHP407-based systems. More in detail, P-CHP407 higher critical deformation could result from the formation of hydrogen bonds between water molecules and the exposed -COOH groups on micelle shell, with water molecules working as “elastic bridges” among adjacent micelles. Furthermore, an additional contribution to the increase in gel critical strain originated from the larger size of P-CHP407 micelles (see Dynamic Light Scattering measurements in the Supporting Information).

#### 3.2.3 pH variation

**Fig. 3:**
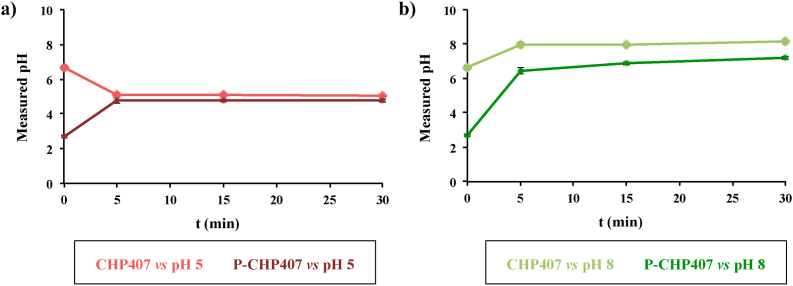
Trend of pH values measured in CHP407 and P-CHP407 gels after 5 min, 10 min, 15 min and 30 min of incubation in contact with buffer at pH 5 (a) and pH 8 (b), at 37 °C.

Chronic wounds are usually associated with bacteria colonization, which contributes to the production of alkaline exudate in the wound bed [[13], [14], [15], [16], [17]]. To investigate the feasibility to exploit this feature as triggering stimulus to enhance the release of system payload, CHP407 and P-CHP407 hydrogel capability to transmit pH changes from the external environment to their core was evaluated by measuring their pH values at different time points upon incubation at 37 °C with phosphate buffers at pH 5 and 8. Although pH measurements were carried out up to 24 h, Fig. 3 reports hydrogel pH variation within the first 30 min of incubation, as the investigated samples were able to completely transfer pH changes from the surrounding environment towards their gel core within this time interval.

Significantly different (*p<0.001*) hydrogel initial pHs were measured for CHP407 and P-CHP407 sol-gel systems, i.e., 6.65 ± 0.13 and 2.69 ± 0.05 respectively, as a consequence of the introduction of carboxylic groups in P-CHP407 chains. Nevertheless, despite these differences and irrespective of the buffer in contact with the samples, both hydrogels were able to change their internal pH very quickly reaching a plateau value within 5 min of incubation. In the case of an acid buffer surrounding the gels (Fig. 3a), CHP407 and P-CHP407 behaved similarly. Indeed, the gap between the hydrogel starting pH and the pH of the acidic buffer was almost the same for both the analyzed systems (ΔpH_CHP407_ = 1.7 *vs.* ΔpH_P-CHP407_ = 2.3). Differently, in the case of alkaline buffer (Fig. 3b), the gap to be covered to reach the pH value of the surrounding buffer was significantly different for the two analyzed systems (ΔpH_CHP407_ = 1.4 *vs.* ΔpH_P-CHP407_ = 5.3). However, although they were both able to acquire the same pH of the buffer within the same time interval, P-CHP407 hydrogels showed an increased sensitivity to basic pH environment, with faster pH variations compared to CHP407-based samples. Indeed, ΔpH within the first 5 min incubation was measured to be 1.32 and 3.76 for CHP407 and P-CHP407 hydrogels, respectively. Therefore, these data successfully proved that the introduction of carboxylic groups could be exploited to provide water-soluble polymers with alkaline pH-responsiveness.

#### 3.2.4 Hydrogel behavior in contact with buffers

**Fig. 4:**
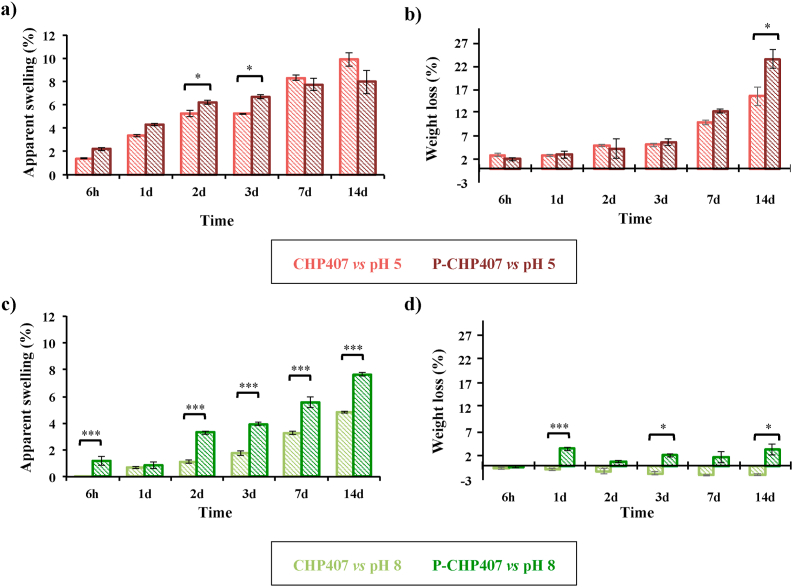
Apparent swelling (a and c) and weight loss (b and d) percentages measured for CHP407- and P-CHP407-based hydrogels in contact with phosphate buffers at pH 5 and 8 for 6 h, 1 day, 2 days, 3 days, 7 days, and 14 days, at 37 °C.

To evaluate hydrogel behavior in pathological-mimicking environments (i.e., inflammation and infection) and to assess their capability to absorb a large amount of exudate, both CHP407 and P-CHP407 hydrogels were incubated in the presence of phosphate buffers at pH 5 or 8 for different time intervals (Fig. 4a–d). Moreover, this test also allowed the investigation of the influence of carboxylic group exposure on hydrogel apparent swelling and dissolution/degradation behaviors.

In acid environment (pH 5) (Fig. 4a and b), both CHP407 and P-CHP407 gels progressively increased their wet weight over time, as a consequence of fluid absorption from the surrounding medium. Up to 3 days of incubation, P-CHP407 gels showed higher apparent swelling percentages compared to CHP407-based samples, with significant differences on day 2 and 3. This behavior can be ascribed to the presence of exposed carboxylic groups along the polymeric chains, which resulted in an increased hydrophilicity. After 7 and 14 days incubation time, the apparent swelling percentages of CHP407-based hydrogels were slightly higher compared to those of P-CHP407 gels (not significant differences). This inversion in the trend is probably correlated to the higher dissolution/degradation degree of P-CHP407 gels with respect to CHP407 ones at these time points (Fig. 4b). Indeed, CHP407 and P-CHP407 gels showed similar weight loss percentages at all the analyzed time points with the only exception of 7 and 14 days incubation times.

Conversely, substantially different trends were observed for hydrogels in contact with an alkaline medium (pH 8) (Fig. 4c and d). In fact, P-CHP407 gels generally showed significantly higher apparent swelling percentages with respect to CHP407 samples, except for the 1 day time point. These remarkably higher values can be correlated to the presence of –COOH groups, which de-protonation at basic pH caused the formation of carboxylate groups and consequent electrostatic repulsion among chains, leading to hydrogel network broadening and fluid absorption. Thus, although both CHP407 and P-CHP407 hydrogels showed high apparent swelling capability, resulting in potentially promising systems for wound management according to the moist wound healing theory [36], P-CHP407 gels were able to absorb significantly higher amounts of alkaline fluids as a consequence of their internal structural changes.

Considering the stability behavior in acid or basic conditions, the two hydrogels showed extremely different results (Fig. 4b and d). Indeed, while a low dissolution/degradation was observed for both systems in alkaline environment up to 14 days, higher weight loss percentages were measured in acid buffer, which increased over time up to approx. 20% on day 14, suggesting that acidic environment strongly destabilized the gel network. An in-depth investigation through chromatographic analyses revealed that prolonged hydrogel incubation with an acidic medium (e.g., 7d, 14d) led to partial polymer degradation, which was significantly more detrimental (*p* < 0.001) for P-CHP407 compared to CHP407 (e.g., 12.2 ± 0.3% and 28.0 ± 2.9% reduction in the initial ${\bar{M}}_{n}$ of CHP407 and P-CHP407 at 14d gel incubation, respectively). Such partial polymer degradation could be probably ascribed to the hydrolytic breaking of ethylene oxide blocks induced by the surrounding acid environment [37]. Thus, the measured weight loss resulted from both hydrogel dissolution and polymer degradation phenomena. Conversely, no significant polymer degradation was observed when systems were in contact with an alkaline buffer (i.e., measured ${\bar{M}}_{n}$ percentage variations within the instrument error [38]).

### 3.3 Hydrogel characterization at the nano-scale through Low Field Nuclear Magnetic Resonance spectroscopy

To thoroughly investigate hydrogel structural changes occurring at the nano-scale in response to external stimuli (i.e., temperature and pH), both CHP407 and P-CHP407 hydrogels were characterized by exploiting the versatility of LF-NMR spectroscopy. Indeed, this non-destructive technique gives information about water organization with respect to polymeric chains by measuring the variation of water hydrogen spin-spin relaxation times (T_2_) upon a very quick change in the orientation of an applied homogeneous magnetic field. Specifically, the permanent dipole of water hydrogens follows the direction of an applied magnetic field and a change in its direction produces a change in the spin orientation. The time spins require to recover initial orientation, directly connected to T_2_, depends on multiple parameters such as temperature and polymeric chain spatial organization; thus, T_2_ variations entail modifications in hydrogel structure. In the case of homogeneous gels, i.e., gels characterized by uniformity in the mesh size distribution, a unique relaxation time would exist. However, in a realistic polymeric system a poly-dispersed mesh size distribution occurs and thus, more than one relaxation time appears. In particular, each *T*_2i_ corresponds to a mesh class characterized by a specific size. The contribution to each *T*_2i_ is represented by the relative abundance parameter *A*_i_, so that the sum of all *A*_i_ values is equal to 1. The weighted average of all the identified contributions defines the mean spin-spin relaxation time T_2m_.

#### 3.3.1 Hydrogel thermo-sensitivity evaluation

During a sol-to-gel transition, T_2m_ generally decreases as a consequence of the spatial re-arrangement of the polymer molecules that give rise to a three-dimensional network upon temperature increase [39]. However, if the formation of the polymeric structure does not imply a clear increase in the portion of the polymeric surface exposed to water molecules, the effect of temperature, which by its nature contributes to T_2m_ increase, cannot be negligible; consequently, the T_2m_ of the overall system can increase. In the presence of micellar gels this effect should be considered, as the formation of a micelle-based network could not necessarily imply a sufficiently high increase of the polymeric surface exposed to water molecules to make the temperature contribution negligible. Furthermore, upon chain arrangement in the form of micelles, the polymeric superficial area, which can interact with water molecules is reduced, resulting in a decrease of bound water and an increase of free water contribution. Hence, in the case of micellar networks, T_2m_ value can increase during the gelation process. To this purpose, both CHP407 and P-CHP407 samples were first equilibrated at 4 °C, then placed at 37 °C and finally, the mean spin-spin relaxation times were recorded as a function of time during this high-temperature equilibration (Table 1).

**Table 1:** Mean spin-spin relaxation times T_2m_ measured for CHP407- and P-CHP407-based systems during equilibration at 37 °C within 10 min.

Mean spin-spin relaxation time T_2m_ (ms)					
	*t* = 0 min	*t* = 4 min	*t* = 6 min	*t* = 8 min	*t* = 10 min
CHP407	1106	1231	1241	1259	1259
P-CHP407	1204	1491	1521	1521	1557

**Fig. 5:**
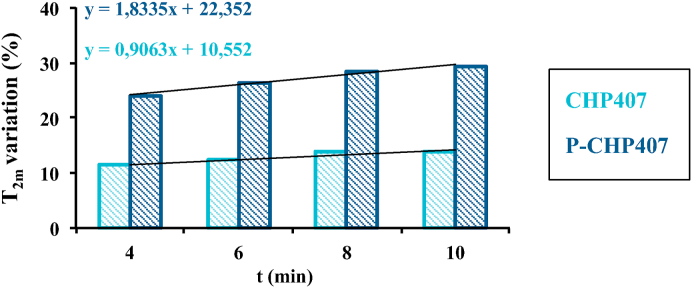
Percentage variation of spin-spin relaxation time (T_2m_) measured as a function of time during equilibration at 37 °C for CHP407- and P-CHP407-based systems.

As argued, T_2m_ values increased as a function of time for both the analyzed systems. However, at each time point, P-CHP407 hydrogels showed higher T_2m_ values with respect to the control. As both samples were subjected to the same heating procedure, differences in T_2m_ as a function of time were ascribed to a different hydrogel organization. Fig. 5 reports the percentage variation (with respect to time zero data) of T_2m_ as a function of time, which resulted faster in P-CHP407 than in CHP407 samples, as highlighted by the tendency line.

This observation suggested that the gelation process started at lower temperatures for P-CHP407 hydrogels in agreement with previously reported temperature ramp test results (Fig. 2a). Such findings can be correlated with the larger size of P-CHP407 micelles with respect to CHP407 ones: bigger micelles result in higher T_2m_ as the magnetic relaxation of water hydrogens trapped in the micelle is less influenced by the polymer.

For what concerns T_2m_ at 10 min of analysis (i.e., systems in the incipient gel state), P-CHP407-based gels showed a higher relaxation time compared to CHP407-based gels, reflecting a different internal structure pertaining to the two systems. Three-dimensional organization of micelles with larger size as in the case of P-CHP407 gave rise to higher void volume among the micelles respect to the case of hydrogels formed by micelles with smaller size as in the case of CHP407. Thus, in the case of P-CHP407 hydrogels, the effect of polymer on T_2m_ was negligible reflecting into higher T_2m_.

#### 3.3.2 Hydrogel pH-responsiveness evaluation

LF-NMR was conducted on CHP407 and P-CHP407 hydrogels upon incubation with buffers at different pH values with the aim to analyze the pH-transmission mechanism through their thickness, i.e., micelle/water molecule interactions and micellar network re-arrangement in response to fluid absorption. To this aim, relaxation time variations were correlated with changes in hydrogel water content, while T_2i_ changes were correlated with water molecule organization. Hydrogel mean spin-spin relaxation times were measured after different incubation times (10 min, 20 min, 18 h and 24 h) in the presence of a buffer at acid or alkaline pH. Due to the different initial T_2m_ value of P-CHP407 and CHP407 hydrogels (i.e., T_2m_ = 1557 ms and 1259 ms, respectively), T_2m_ percentage variation respect to the time zero measurement was calculated at each investigated time point (Table 2).

**Table 2:** T_2m_ percentage variation with respect to its time zero value for CHP407 and P-CHP407 hydrogels after incubation with phosphate buffer at pH 5 or 8 for 10 min, 20 min, 18 h and 24 h at 37 °C.

Δ% T_2m_*vs.* pH 5 (%)				
	*t = 10 min*	*t = 20 min*	*t = 18 h*	*t = 24 h*
CHP407	18.1	6.3	13.2	49.0
P-CHP407	6.3	−1.2	8.4	19.4
**Δ% T**_**2m**_***vs.*****pH 8 (%)**				

	*t = 10 min*	*t = 20 min*	*t = 18 h*	*t = 24 h*

CHP407	−1.8	−3.2	−1.5	3.0
P-CHP407	−2.0	2.1	−0.7	10.2

Hydrogels incubated in alkaline buffer did not show any significant change in T_2m_ up to 18 h, suggesting that their hydrogel structure remained almost unchanged. The only exception was observed for P-CHP407-based systems after 24 h: a remarkably higher T_2m_ variation was detected for P-CHP407 hydrogels compared to CHP407 ones (10.2% *vs.* 3.0%, respectively), confirming that P-CHP407 hydrogels increased their water content in the presence of an alkaline buffer, in agreement with swelling test results. The improved ability of P-CHP407 hydrogels to modify their structure, absorbing a higher amount of buffer with alkaline pH, was a consequence of the presence of carboxylic groups. In fact, in an alkaline environment, -COOH groups become deprotonated and exposed negative charges attract a higher amount of hydronium ions through electrostatic interactions.

To thoroughly investigate changes in water organization and interaction with micelles inside the hydrogels, T_2i_ values and their corresponding A_i_ were analyzed and reported in Table 3, Table 4*.* Specifically, results are reported as percentage variations of each T_2i_ referred to the corresponding time zero value, and fold change of their corresponding participation percentage A_i_ with respect to the initial value (Table S1).

**Table 3:** Variations of each T_2i_ component expressed as % and referred to the time zero value, and fold change of each participation percentage A_i_ with respect to the initial value for CHP407 and P-CHP407 hydrogels in contact with phosphate buffer at pH 8 for 10 min, 20 min, 18 h and 24 h, at 37 °C.

	CHP407 *vs.* pH 8					
	Δ%T_21_ (%)	Fold A_1_	Δ%T_22_ (%)	Fold A_2_	Δ%T_23_ (%)	Fold A_3_
10 min	−1.5	1.0	15.2	1.1	25.5	1.0
20 min	−3.3	1.0	4.2	1.0	12.8	1.0
18 h	−2.4	1.0	−5.1	1.0	6.0	0.9
24 h	3.7	0.9	53.0	1.5	97.2	1.1

	**P-CHP407*****vs.*****pH 8**					

	Δ%T_21_ (%)	Fold A_1_	Δ%T_22_ (%)	Fold A_2_	Δ%T_23_ (%)	Fold A_3_

10 min	−0.5	1.0	8.2	1.2	10.8	1.0
20 min	3.1	1.0	9.5	1.1	13.3	1.0
18 h	1.1	1.0	16.9	1.3	7.3	1.0
24 h	22.5	0.7	83.3	3.1	75.3	1.2

**Table 4:** Variations of each T_2i_ component expressed as % and referred to the time zero value, and fold change of each participation percentage A_i_ with respect to the initial value for CHP407 and P-CHP407 hydrogels in contact with phosphate buffer at pH 5 for 10 min, 20 min, 18 h and 24 h, at 37 °C.

CHP407 *vs.* pH 5						
	Δ%T_21_ (%)	Fold A1	Δ%T_22_ (%)	Fold A_2_	Δ%T_23_ (%)	Fold A_3_
10 min	13.5	1.0	12.5	1.0	3.6	0.8
20 min	5.5	1.0	−10.6	0.9	−19.5	0.9
18 h	12.0	1.0	40.7	1.1	56.3	0.9
24 h	41.2	1.1	5.8	0.9	–	–
**P-CHP407*****vs.*****pH 5**						
	Δ%T_21_ (%)	Fold A1	Δ%T_22_ (%)	Fold A_2_	Δ%T_23_ (%)	Fold A_3_
10 min	3.3	1.1	−1.5	0.8	−1.8	0.9
20 min	0.6	1.0	16.9	1.3	14.4	1.1
18 h	14.0	0.8	62.0	2.2	56.6	1.1
24 h	17.2	1.0	45.1	1.0	39.7	0.9

Irrespective of the tested buffer, the presence of three different T_2i_ evidences that water hydrogens inside the gel structure contribute to the final relaxation time T_2m_ with three different fractions.

Concerning samples in contact with the alkaline buffer, although P-CHP407 hydrogels did not show any variation in T_2m_ after 18 h of incubation (Table 2 - Δ%T_2m_ *vs.* pH 8), significant changes were observed in the variation of each component T_2i_ (Table 3). Specifically, while T_21_, T_23_ and their corresponding participation percentages remained almost constant, T_22_ was subjected to a considerable variation, with an increase of its value and its corresponding participation percentage (16.9% and 1.3-fold, respectively). These observations suggested that pH-triggered changes in water molecule organization occurred within the hydrogel.

**Fig. 6:**
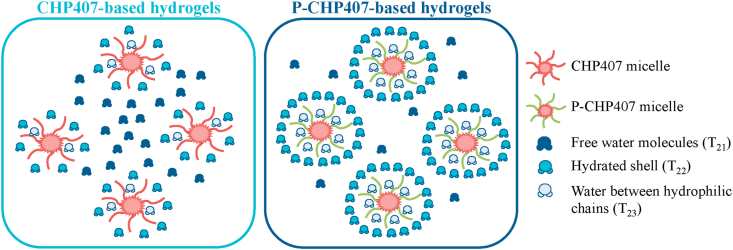
Schematic representation of water molecules interaction with CHP407- and P-CHP407-based micelles. Water molecules between the hydrophilic chains contribute to the determination of T_23_, those around micelles contribute to the determination of T_22_ and those between micelles contribute to the determination of T_21_.

Based on the absolute values of T_21_, T_22_ and T_23_ (Table S1), and the theoretical principles underpinning LF-NMR characterization, we could suppose that T_23_ refers to water molecules present between micelle hydrophilic chains, T_22_ characterizes the kind of water which forms a hydrated shell around micelles and T_21_ is the relaxation time of water molecules in the interstitial space between micelles (Fig. 6). Hence, an increase in the T_22_ component for P-CHP407 hydrogels is in agreement with the hypothesis that the presence of carboxylic groups favors the formation of a hydrated shell around its micelles through electrostatic interactions. This hypothesis was further confirmed by the unchanged values of T_2i_ components for CHP407-based hydrogels up to 18 h (Table 3).

After 24 h of incubation with alkaline buffer, T_2m_ increased for both systems (Table 2 - Δ%T_2m_ *vs.* pH 8), but T_2i_ changes were extremely different between the two analyzed hydrogels (Table 3). In detail, T_2m_ increase in CHP407-based gels was mainly due to an increase in the T_22_ component (i.e., 53% - water molecules surrounding the micelles), while T_2m_ increase in P-CHP407-based gels was the result of an increase of T_21_, T_22_ and T_23_. Specifically, P-CHP407 hydrogels showed 75.3% increase of T_23_ (i.e., water electrostatically interacting with hydrophilic domains in micelle interstitial space), and a slight variation of its participation A_23_ (only 1.2-fold the initial value), justified by the small available space for water molecules. However, for P-CHP407 hydrogels, the main contribution in T_2m_ change was due to the huge increase of T_22_ with a variation of 83.3% compared to the initial value, and A_22_ participation being 3.1-fold respect to the initial value. This result confirmed the ability of carboxylic groups to form a more consistent hydrated shell around each P-CHP407 micelle, compared to CHP407 gels, where water molecules only interacted with micelles through polymer hydrophilic building blocks. Finally, for P-CHP407 hydrogels, the increase of T_21_ (i.e., water molecules in the interstitial space among micelles) compared to CHP407 samples confirmed swelling test results. Indeed, P-CHP407 hydrogels showed higher capability to absorb water from the surrounding environment with respect to CHP407 hydrogels, thus suggesting their promising application in the treatment of hard-to-close wounds. However, while A_21_ for CHP407 hydrogel was 0.9-fold higher than the initial one, A_21_ for P-CHP407 gel was slightly lower, as in this second case the absorbed fluid mainly interacted with carboxylic groups rather than remaining in the interstitial space among micelles.

Concerning hydrogel incubation with a phosphate acidic buffer, the measured T_2m_ values showed substantial differences respect to initial T_2m_ values (Table 2), even after only 10 min incubation in the acidic medium (Table 2 - Δ%T_2m_ *vs.* pH 5). Furthermore, a comparison of T_2m_ values at fixed times showed that CHP407-based gels were able to absorb a higher amount of water from the surrounding environment respect to P-CHP407 hydrogels. These results were in agreement with hydrogel behavior in contact with a buffer at pH 5, which showed significantly higher weight loss percentages compared to alkaline conditions. Hence, the higher values of T_2m_ were attributed to an overall higher amount of water taking part to hydrogel dissolution. This hypothesis was further confirmed by the evaluation of T_2i_ variations as a function of time (Table 4) showing unchanged participation percentages, measured at each time point, suggesting water uptake by samples and their partial dissolution.

### 3.4 pH-controlled drug release test

**Fig. 7:**
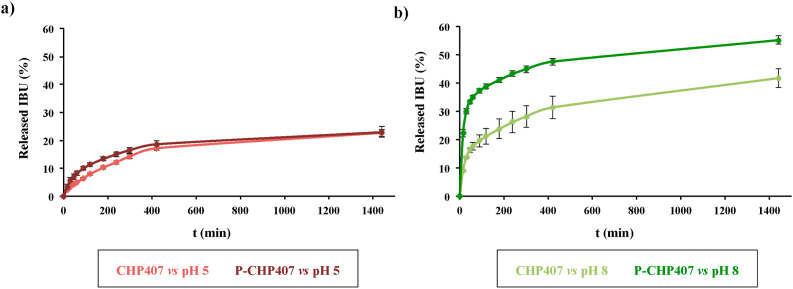
Released IBU (expressed as percentage with respect to the total encapsulated amount) from CHP407 and P-CHP407 hydrogels in contact with phosphate buffers at pH 5 (a) and 8 (b).

To definitely prove the hydrogel capability to respond to external pH changes, thus releasing their payload via a pH-controlled mechanism, drug release tests were conducted on both CHP407- and P-CHP407 gels in contact with phosphate buffers at pH 5 (i.e., inflammatory-mimicking exudate pH) or 8 (i.e., infected-mimicking exudate pH) (Fig. 7). Among available drugs in the market, Ibuprofen (IBU) was selected as model molecule because it is widely used as anti-inflammatory and analgesic drug in the treatment of chronic wounds, which are characterized by an alkaline exudate [[40], [41], [42]]. Despite IBU hydrophobic nature, which could potentially hinder drug loading into a hydrophilic network, we recently reported on the possibility to exploit the PEU micellar organization to encapsulate drugs with different wettability [43]. Specifically, we observed that hydrophobic molecules like IBU can be entrapped in the hydrophobic micelle core during chain arrangement into spherical structures. Conversely, hydrophilic molecules can be entrapped in the interstitial spaces among micelles. Thus, starting from this knowledge, in this work we selected 1 mg/mL IBU concentration, in accordance with our previous work on the design of CHP407-based hydrogels as delivery systems [44], and we investigated PEU hydrogel potentialities as smart pH-controlled drug vehicles.

When sol-gel systems were in contact with the acidic buffer no significant differences were observed in the released amount of IBU from CHP407 or P-CHP407 gels at each analyzed time point. Hence, as expected, -COOH groups did not play an active role in drug release mechanism in an acid environment. Conversely, these evidences suggested that the payload release was the result of the high swelling/dissolution degree [7].

These observations were further supported by the macroscopic description of the release kinetics according to the Korsmeyer-Peppas equation within the first 7 h of incubation (being this model valid up to the 60% released drug). This equation gave for both systems an *n* value characteristic of a macroscopic non-Fickian anomalous transport (i.e., 0.61 ± 0.20 and 0.52 ± 0.12 for CHP407 and P-CHP407 systems, respectively) [35].

On the other hand, when hydrogels were in contact with an alkaline medium, an extremely different macroscopic drug release kinetics was observed. Specifically, the released amount of IBU from P-CHP407 hydrogels was significantly higher (*p* < 0.0001) with respect to the control at each analyzed time point. Considering that both CHP407 and P-CHP407 gels showed higher resistance against dissolution/degradation when incubated in basic environment compared to acidic medium (Fig. 4d), the different drug release kinetics was probably not ascribed to hydrogel dissolution phenomena. This suggestion was further supported by the application of the Korsmeyer-Peppas model, with an *n* value characteristic of the macroscopic Fickian diffusion transport mechanism (i.e., 0.37 ± 0.02 and 0.16 ± 0.01 for CHP407 and P-CHP407 gels in contact with pH 8 buffer, respectively) [35]. Therefore, at each time point, the extremely different amounts of released drug from CHP407 and P-CHP407 gels against an alkaline medium could be ascribed to the presence of carboxylic acid groups along P-CHP407 chains. Specifically, upon deprotonation, carboxylic groups in P-CHP407 hydrogels formed carboxylate groups which caused electrostatic repulsion among the polymeric chains, increasing hydrogel mesh size with consequent more rapid drug release. Hence, the presence of exposed carboxylic groups could effectively trigger the release of hydrogel payload through the absorption of alkaline medium.

On the other hand, the absence of pH-responsive groups in CHP407 hydrogels should result in similar drug release profiles irrespective of the medium pH (i.e., pH 5 and 8). However, slightly higher IBU amounts were released in an alkaline medium compared to acid one. These observations could not be ascribed to hydrogel behavior in watery medium, as CHP407 hydrogels showed lower weight loss percentages at alkaline pH with respect to acid one (Fig. 4b and d). Conversely, they could be explained considering the pH-dependent IBU solubility, which was measured to be significantly lower at pH 5 with respect to pH 8 (i.e., 0.28 mg/mL *vs.* 3.51 mg/mL at 37 °C) [33].

### 3.5 *In vitro* hydrogel cytocompatibility

To investigate hydrogel cytocompatibility, Resazurin viability assay was conducted on both CHP407 and P-CHP407 extracts using NIH-3T3 murine fibroblasts according to the ISO 10993 regulation. Results showed no statistically significant differences in cell viability for both hydrogels compared to the control. In more detail, 96 ± 5.8% and 88.3 ± 8.5% percentage viability was measured for CHP407 and P-CHP407 hydrogels, respectively, indicating the absence of cytotoxic by-product release. Biocompatibility of P-CHP407 hydrogels confirmed their potential use as smart systems for drug delivery in the advanced treatment of chronic infected wounds.

## 4 Conclusion

In this work, the design of a smart drug delivery system able to ensure a temperature-driven drug loading and a pH-controlled drug release was successfully carried out with the aim to provide a powerful tool for the advanced treatment of chronic skin wounds. Specifically, exploiting the amphiphilic nature of the macrodiol and the exposure of carboxylic groups (i.e., 8.8 ± 0.9 nmol/g_polymer_) through plasma treatment, the synthesized poly(ether urethane) was able to provide hydrogels with a double responsiveness (i.e., temperature and alkaline pH, respectively). The influence of functional group exposure on hydrogel thermo-responsiveness was first qualitatively assessed through Tube Inverting and Gelation Time tests. Moreover, these tests allowed the selection of a promising hydrogel formulation (i.e., 15% w/V PEU concentration) for application as injectable drug delivery system. Then, an in-depth investigation was carried out through rheological characterization, showing that the acidic groups played an active role in the gelation process. Specifically, their presence in P-CHP407 shifted the gelation onset to lower temperatures as they caused the formation of micelles with larger size than for CHP407. However, their slow assembly into an organized network slowed down the overall gelation kinetics. These observations were further corroborated by the nano-scale characterization through LF-NMR: T_2m_ values suggested the presence of polymeric structures strongly interacting with the surrounding water molecules. On the other hand, hydrogel pH-responsiveness was studied through the measurement of the pH variation in the gel core upon incubation in the presence of buffers with different pHs (i.e., 5 and 8 simulating different pathological wound phases) and through swelling/stability tests. P-CHP407 gels showed a significantly higher capability to transmit alkaline pH changes from the surrounding environment to the gel core through the electrostatic repulsion occurring between polymeric chains caused by carboxylic group deprotonation in an alkaline medium. Moreover, these observations were further supported by a significantly higher apparent swelling capability of P-CHP407 gels incubated in basic medium compared to CHP407 ones. Furthermore, LF-NMR spectroscopy further proved at the nano-scale that carboxylic groups in P-CHP407 led to tighter interactions with water molecules with respect to CHP407. Concerning acid pH, no significant differences were observed both at the macro- and nano-scale between CHP407 and P-CHP407 hydrogels in terms of pH transmission capability and hydrogel behavior in watery medium. In addition, both systems showed high cell viability according to the ISO 10993 regulation. Finally, hydrogel capability to release their payload (e.g., Ibuprofen) via a pH-triggered mechanism was assessed through drug release tests, showing a significantly higher cargo release from P-CHP407 gels compared to CHP407 systems upon incubation in alkaline milieu.

Therefore, starting from an *ad hoc* synthesized and functionalized polymeric component [26], the here-developed hydrogel was able to successfully encapsulate an anti-inflammatory drug and release its payload via a pH-controlled mechanism. The demonstration of the hydrogel capabilities thus paves the way to the design of drug carriers with improved therapeutic efficiency. Moreover, hydrogel injectability [30] and thermo-responsiveness favor drug loading and the use of the resulting formulation as an *in situ* drug-delivery injectable hydrogel for the more effective treatment of hard-to-close skin wounds.

## CRediT authorship contribution statement

**Rossella Laurano:** Conceptualization, Methodology, Validation, Formal analysis, Investigation, Data curation, Writing - original draft, Visualization. **Monica Boffito:** Conceptualization, Methodology, Validation, Investigation, Writing - review & editing, Supervision. **Michela Abrami:** Investigation, Data curation, Writing - review & editing. **Mario Grassi:** Supervision, Writing - review & editing. **Alice Zoso:** Investigation, Data curation, Writing - review & editing. **Valeria Chiono:** Supervision, Writing - review & editing. **Gianluca Ciardelli:** Resources, Supervision, Funding acquisition, Writing - review & editing.

## Declaration of competing interest

The authors declare no competing financial interest.
