# Supplementary material for: Dual stimuli-responsive polyurethane-based hydrogels as smart drug delivery carriers for the advanced treatment of chronic skin wounds
Source: Bioact Mater. 2021 Feb 19;6(9):3013–24. doi: 10.1016/j.bioactmat.2021.01.003 (PMC8233160; doi:10.1016/j.bioactmat.2021.01.003)
Supplement: Multimedia component 1 [file mmc1.docx]

**Dual stimuli-responsive polyurethane-based hydrogels as smart drug delivery carriers for the advanced treatment of chronic skin wounds**

Rossella Laurano^a,§^, Monica Boffito^a,§,*^, Michela Abrami^b^, Mario Grassi^b^, Alice Zoso^a^, Valeria Chiono^a^, Gianluca Ciardelli^a^

^a^ Politecnico di Torino, Mechanical and Aerospace Engineering Department, Corso Duca degli Abruzzi 24, 10129 Torino, Italy (R.L. [rossella.laurano@polito.it](mailto:rossella.laurano@polito.it); M.B. [monica.boffito@polito.it](mailto:monica.boffito@polito.it); A.Z. [alice.zoso@polito.it](mailto:alice.zoso@polito.it); V.C. [valeria.chiono@polito.it](mailto:valeria.chiono@polito.it); G.C. [gianluca.ciardelli@polito.it](mailto:gianluca.ciardelli@polito.it))

^b^ Università degli Studi di Trieste, Department of Engineering and Architecture, Via Alfonso Valerio 6/1, 34127, Trieste, Italy (M.A. [mikystars@hotmail.com](mailto:mikystars@hotmail.com); M.G. [mario.grassi@dia.units.it](mailto:mario.grassi@dia.units.it))

*Chemical characterization of the synthesized and plasma-treated poly(ether urethane)*

Both the synthesized (i.e., CHP407) and the plasma-treated (i.e., P-CHP407) poly(ether urethane)s (PEUs) were chemically characterized through Attenuated Total Reflectance Fourier Transform Infrared spectroscopy, Size Exclusion Chromatography and Toluidine Blue O colorimetric assay by adopting the methods reported by Laurano and Boffito at al. [Laurano, R. and Boffito, M. et al. (2020) Polymers, 11, 2109-2126].

Methods

Attenuated Total Reflectance Fourier Transform Infrared spectroscopy. Attenuated Total Reflectance Fourier Transform Infrared (ATR-FTIR) spectroscopy was first conducted on the synthesized CHP407 as such and on Poloxamer® 407 (P407) macrodiol to prove the success of the synthesis through the appearance of the peaks ascribed to the newly formed urethane bonds. Then, the same analyses were also performed on plasma-treated PEU powders and results compared to CHP407 spectrum as control condition. Specifically, analyses were conducted in triplicate at room temperature using a Perkin Elmer Spectrum 100 equipped with an ATR accessory (UATR KRSS) with diamond crystal. Spectra were recorded in the spectral range from 4000 to 600 cm^-1^ (resolution 4 cm^-1^, 32 scans), analyzed using the Perkin Elmer Spectrum software and reported as average profiles.

Size Exclusion Chromatography. Size Exclusion Chromatography (SEC) analyses were performed on both CHP407 and P-CHP407 samples to assess potential degradation phenomena induced by plasma treatment. The instrument (Agilent Technologies 1200 Series, Agilent Technologies Inc., USA) was equipped with a Refractive Index (RI) detector and two Waters Styragel columns (HR1 and HR4) conditioned at 55 °C. Polymers (2 mg) were first dissolved in 1 mL of mobile phase (eluent flow: 0.5 mL/min), i.e., N,N-dimethylformammide (CHROMASOLV Plus, inhibitor free, for HPLC, 99.9%, Carlo Erba Reagents, Italy) added with 0.1% w/V lithium bromide (LiBr, Sigma Aldrich, Italy); then, solutions were filtered through a 0.45 μm syringe filter (poly(tetrafluoroethylene) membrane, Whatman). Number Average Molecular Weight ($\bar{M}_{n}$), Weight Average Molecular Weight ($\bar{M}_{w}$) and Polydispersity Index (D) were estimated using the Agilent ChemStation software starting from a calibration curve based on poly(ethylene glycol) standards with Peak Molecular Weight in the 1000 – 200000 Da range. Analyses were performed in triplicate and results were reported as average profiles or mean values ± standard deviation.

Toluidine Blue O colorimetric analysis. Carboxylic acid groups exposed by means of plasma treatment were colorimetrically quantified through Toluidine Blue O (TBO) assay. In detail, 20 mg of P-CHP407 powders were first dissolved in 500 μM TBO (Sigma Aldrich, Italy) aqueous solution (40 mL, pH 10) and then the coupling reaction between cationic dye molecules and deprotonated carboxylic groups was carried on for 12 hours at room temperature. Subsequently, samples were put in dialysis (cut off membrane 10 – 12 kDa, Sigma Aldrich, Italy) against double distilled water to remove unreacted TBO molecules, and finally freeze-dried (Martin Christ ALPHA 2-4 LSC, Germany). Then, 10 mg of freeze-dried samples were dissolved in acetic acid/double distilled water (1 mL, 50/50 V/V) for 30 min to allow the desorbing reaction of bonded/adsorbed TBO molecules. Finally, the polymer was separated by centrifugation at 10000 rpm, 15 °C for 10 min and the extract absorbance was measured at 632 nm using an UV/Vis spectrophotometer (PerkinElmer, Lambda 25). CHP407 samples were treated according to the same procedure as control condition. Carboxylic acid groups were quantified by referring to a calibration curve based on TBO molecules dissolved in the desorbing medium at different concentrations (1 – 20 μM range). Analyses were performed in triplicate and results were reported as average absorbance profiles and mean value ± standard deviation.

Results

Attenuated Total Reflectance Fourier Transform Infrared spectroscopy

**Figure S1.** ATR-FTIR spectra of P407 (orange spectrum) and the synthesized poly(ether urethane) CHP407 (light blue spectrum). The bands proving the success of the synthesis are highlighted in bold.

**Figure S2.** Comparison between the ATR-FTIR spectra of CHP407 (light blue) and P-CHP407 (dark blue) samples. The bands ascribed to the urethane bonds are highlighted in bold, while the peaks proving the successful exposure of carboxylic groups are reported in red.

**Size Exclusion Chromatography

**Figure S3.** Molecular weight distribution profiles (normalized refractive index -RI- *vs.* molecular weight of each chain composing the sample -M_i_-) of un-treated (CHP407 – light blue profile) and plasma-treated (P-CHP407 – dark blue profile) poly(ether urethane) powder. The peak of P-CHP407 molecular weight distribution profile slightly moved to lower molecular weights compared to CHP407 one. However, considering the instrument error (i.e., 10% according to Trathnigg, B. [Trathnigg, B. (2000) Encyclopedia of Analytical Chemistry, 8008-8034] no statistically significant differences were registered in terms of average values between un-treated and plasma-treated poly(ether urethane)s (e.g., Number Average Molecular Weight was measured to be 34.0 ± 1.3 and 29.0 ± 0.8 kDa for CHP407 and P-CHP407, respectively).

Toluidine Blue O colorimetric analysis

**Figure S4.** Toluidine Blue O (TBO) colorimetric assay performed on un-treated (CHP407 – light blue) and plasma-treated (P-CHP407 – dark blue) poly(ether urethane) powders. a) Representative image of CHP407 and P-CHP407 solutions upon interaction with TBO molecules: light and dark blue colors mark adsorbed and electrostatically bonded dye molecules to polymeric chains, respectively. b) TBO absorbance intensity profiles measured for CHP407 and P-CHP407 after 30 min of incubation in the desorbing medium. The red dashed line marks the TBO maximum absorbance wavelength (i.e., 632 nm).

*Qualitative evaluation of sol-to-gel transition*

**

**Figure S5.** Representative images of the temperature-driven sol-to-gel transition of un-treated and plasma-treated poly(ether urethane)-based systems: sol state at 4 °C (left) and gel state (right) upon incubation at 37 °C for 10 minutes.

*Dynamic Light Scattering*

Method

To investigate changes in micelle hydrodynamic diameters induced by the exposure of carboxylic acid groups along plasma-treated polymeric chains, Dynamic Light Scattering (DLS) measurements were performed on both CHP407 and P-CHP407 formulations. In detail, polymers were first dissolved at 0.1% w/V concentration in physiological saline solution (0.9% NaCl) and then, analyses were conducted at physiological temperature using a Zetasizer Nano S90 (Malvern Instruments, Worcestershire, UK) instrument. Before starting, formulations were equilibrated at 37 °C for 15 minutes and then analyzed according to Pradal et al. [Pradal, C. et al. (2013) Biomacromolecules 14, 3780-3792]. Analyses were performed in triplicate and the average hydrodynamic diameters were reported as mean values ± standard deviation.

Results

Dynamic Light Scattering (DLS) analyses proved that both poly(ether urethane)-based formulations were able to form micellar structures at physiological temperature due to the presence of Poloxamer® 407 as chain building block [Laurano, R. and Boffito, M. (2020) Front. Bioeng. Biotechnol. 8:708; Boffito, M. et al. (2016) Polym. Int. 65:756; Laurano, R. et al. (2020) React. Funct. Polym. 146:104413]. However, the distribution pattern by volume of P-CHP407 micelle average hydrodynamic diameters was shifted to higher values compared to that of CHP407 one, thus proving the formation of larger micelles in P-CHP407-based samples at body temperature. More in detail, the average hydrodynamic diameter was measured to be 32.3 ± 1.3 nm and 36.1 ± 1.9 nm for CHP407 and P-CHP407 micelles, respectively. Therefore, as the polymeric component of the analyzed systems belonged to the same synthesis procedure, such differences can be mainly attributed to the exposure of carboxylic acid groups upon plasma treatment. Indeed, these functional groups are able to strongly interact with the surrounding water molecules leading to the formation of a hydrated shell around micelles and thus, to the achievement of larger structures.

**

**Figure S6.** Distribution pattern by volume of micelle hydrodynamic diameters measured for CHP407 (light blue) and P-CHP407 (dark blue) formulations (0.1% w/V polymeric concentration) at physiological temperature.

*Strain Sweep test at 37 °C*

**Figure S7.** Storage (G’) and loss (G”) moduli recorded as a function of the applied deformation within the range 0.01% - 500% for CHP407 (light blue) and P-CHP407 (dark blue).

*Low Field Nuclear Magnetic Resonance (LF-NMR) spectroscopy*

**Table S1.** T_2i_ and the corresponding A_i_ values measured for CHP407 and P-CHP407 systems in the gel state before incubation with buffers (time zero).

|  | T_21_ (ms) | A_1_ | T_22_ (ms) | A_2_ | T_23_ (ms) | A_3_ |
| --- | --- | --- | --- | --- | --- | --- |
| CHP407 | 1401.0 | 84.6 | 513.6 | 9.4 | 73.5 | 6.0 |
| P-CHP407 | 1751.4 | 81.1 | 681.5 | 11.0 | 117.8 | 7.9 |
